# MRI thresholds for discrimination between normal and mild temporomandibular joint involvement in juvenile idiopathic arthritis

**DOI:** 10.1186/s12969-015-0051-7

**Published:** 2015-12-01

**Authors:** Grace Mang Yuet Ma, Afsaneh Amirabadi, Emilio Inarejos, Mirkamal Tolend, Jennifer Stimec, Rahim Moineddin, Lynn Spiegel, Andrea S. Doria

**Affiliations:** Department of Radiology, Ohio State University Wexner Medical Center, 410 West 10th Ave, Columbus, OH 43210 USA; Department of Diagnostic Imaging, The Hospital for Sick Children, 555 University Avenue, Toronto, ON M5G1X8 Canada; Department of Diagnostic Imaging, Hospital Sant Joan de Deu, Passeig Sant Joan de Déu, 2, 08950 Esplugues de Llobregat, Barcelona, Spain; Department of Family and Community Medicine, University of Toronto, 500 University Ave, Toronto, ON M5G 1V7 Canada; Department of Rheumatology, The Hospital for Sick Children, 555 University Avenue, Toronto, ON M5G1X8 Canada

**Keywords:** MRI, Thresholds, Juvenile idiopathic arthritis, Temporomandibular joints, Children

## Abstract

**Background:**

Currently there is no consensus agreement on the degree of enhancement in normal temporomandibular joints (TMJ) in children, which makes it difficult for clinicians to distinguish between the presence/absence of mild synovitis. Quantitative measurements of synovial and condylar enhancement may be useful additions to current qualitative methods on early MRI diagnosis and follow up of TMJ involvement in JIA. The purpose of the study is to establish thresholds/tendencies for quantitative measures that enable distinction between mild TMJ involvement and normal TMJ appearance based on the degree of synovial and bone marrow enhancement in JIA patients.

**Methods:**

TMJ MRI examinations in 67 children with JIA and in 24 non-rheumatologic children who underwent MRI for neurologic/orbit indications were retrospectively assessed. As a priori determined TMJs of JIA patients were categorized into three groups by experienced staff radiologists based on the degree of synovial and condylar enhancement: no active disease (rheumatologic control), mild and moderate/severe findings. The signal intensity (SI) of the synovial tissue around each condyle and of the bone marrow was measured to calculate the enhancement ratio (ER) and relative SI change. The ER was calculated using signal to noise ratios, while relative SI change was calculated using signal intensities alone. Quantitative measurements of synovial and condylar enhancement of TMJs with mild or moderate/severe findings were compared with the rheumatologic and non-rheumatologic controls.

**Results:**

Mean ER values were significantly different between the TMJs without active disease and those with mild and moderate/severe synovial enhancement, with highest values in the moderate/severe group (*P < 0.0001*). Similar findings were seen for condylar enhancement with *P < 0.005*. Relative SI change was unable to differentiate TMJs with mild synovitis from the two controls (*P > 0.10*). 27/60 (45 %) TMJs without active disease had osteochondral changes. 8/40 (20 %) TMJs in the mild group did not demonstrate any synovial thickening.

**Conclusions:**

Quantitative signal to noise ratios of TMJ synovial and condylar enhancement generate thresholds/tendencies, which offer additional information to differentiate mild synovitis from normal TMJs in JIA patients. Osteochondral changes and synovial thickening may not be reliable indicators of active TMJ involvement and should be differentiated from synovial enhancement.

## Background

Juvenile idiopathic arthritis (JIA) is the most common rheumatologic disease of childhood [1]. The temporomandibular joint (TMJ) is commonly involved in children and adolescents with a documented prevalence as high as 75 % [2–4]. Due to its unique anatomy with the mandibular growth plate lying just under a thin layer of fibrocartilage at the surface of the condylar head, the TMJ is particularly susceptible to damage [5]. As a result, early detection and treatment is critical in preventing severe growth disturbances, which would lead to significant facial and joint deformities [2, 6–9]. However, early detection and monitoring is often difficult due to the “silent” (asymptomatic) nature of TMJ arthritis [4]. Consequently, imaging modalities play a vital role in the diagnosis and early treatment of TMJ arthritis in JIA patients [6, 10].

MRI with contrast enhancement is considered the reference standard for evaluating early signs of TMJ inflammation, which includes synovial enhancement, synovial hypertrophy, joint effusion and condylar enhancement [3, 7, 11]. TMJ synovitis has been previously graded as absent, mild, moderate/severe based on the degree of synovial enhancement on MRI [12]. However, to the best of our knowledge, there is currently no consensus agreement on the degree of synovial enhancement that defines mild TMJ arthritis as compared to normal enhancement in children. This often makes it difficult for clinicians to distinguish between the presence and absence of mild synovitis. The decision to institute or discontinue therapy in centers, where intra-articular corticosteroid injections are used, depends on an accurate MRI diagnosis of TMJ inflammation [13]. A recent study by Von Kalle et al. [14] evaluated the degree of contrast enhancement of TMJ synovial tissues in JIA and non-TMJ affected controls using dynamic contrast-enhanced MRI. Although it is important to learn about the patterns of time-intensity curves on JIA TMJs, they require post-processing, which is not always feasible during clinical practice assessments. On the other hand, further clarification on static contrast-enhanced MRI is needed before it may be regarded as the reference standard measure in the assessment of the TMJ, as previous literature mainly relied on subjective visual assessment of joint enhancement [11, 15]. To our knowledge, previous studies have described qualitative methods for accessing the degree of synovial and condylar enhancement [11, 15], but few if any studies have described both qualitative (synovial thickness, enhancement and condylar enhancement) and quantitative (synovial and condylar signal intensity) methods of assessment of TMJ inflammation in the pediatric population. In this retrospective study we used not only the non-affected TMJs of non-rheumatologic subjects as controls (whose examinations are prone to inconsistencies related to technique and gadolinium injection timing), but also the TMJs of JIA patients without any evidence of active inflammation on MRI.

The purpose of this study was to determine the feasibility of subjective and objective measures to distinguish between the various degrees of TMJ inflammation using quantitative measurements of synovial and condylar enhancement. Specifically, to establish thresholds/tendencies for quantitative signal intensity measures on static contrast-enhanced MRI that could enable distinction between mild TMJ involvement and normal TMJ appearance based on the degree of synovial and condylar enhancement in JIA patients.

## Methods

### Patients

This retrospective study was approved by the Research Ethics Board at The Hospital for Sick Children and is Health Insurance Portability and Accountability Act (HIPAA) compliant.

The study design was based on the review of TMJ MRI examinations performed at our institution between January 1^st^ 2010 to December 31^st^ 2013 from three groups: (1) TMJs with evidence of inflammation on MRI in patients under the age of 18 diagnosed with JIA according to the International League of Associations for Rheumatology (ILAR) criteria [16] (rheumatologic cases); (2) TMJs without MRI evidence of active inflammation in the aforementioned JIA population (rheumatologic controls); and (3) TMJs of children without any rheumatologic disease who had contrast enhanced MRI of the brain (non-rheumatologic controls).

To obtain a list of patients for our non-rheumatologic control, patients with at least one MRI of the brain were retrieved from the Picture Archiving and Communication System (PACS) at our institution. Only patients with brain MRI examinations containing images in both the coronal and axial planes were included in the study. Children with unavailable clinical indications for the examination or vascular malformations were excluded from the study due to their potential to increase blood flow into the TMJ. The clinical indications for performance of brain MRI examinations in our non-rheumatologic controls are available in Table 1.

**Table 1:** Indications for MRI brain in non-rheumatologic controls

Developmental delay	
Infection	
Orbital inflammatory syndrome	
Nystagmus	
Optic pathway glioma	
Pituitary adenoma	
Langerhan cell histocytosis	
Brain tumor (excluding vascular lesions)	
Ganglioglioma	
Metastatic disease	
New onset seizures	
Nasal dermoid	

In terms of our rheumatologic cases and controls, patients with at least one MRI of the TMJ within the study period were retrieved from our institutional PACS. Again, only patients with MRI examinations containing images in both the coronal and axial planes were included in the study. The clinical characteristics of our JIA patients were obtained from their medical records and are listed in Table 2. Patients with unavailable clinical data or without an underlying diagnosis of JIA according to the ILAR criteria [16] were excluded from the study.

**Table 2:** Baseline characteristics of patients with juvenile idiopathic arthritis (JIA) and non-rheumatologic controls. Osteochondral changes are defined as one or more of the following: articular surface erosions, subchondral cysts or condylar flattening

	JIA with/without active TMJ involvement				Non-rheumatologic
	All	Unilateral	Bilateral	Both TMJs unaffected	-
# of patients, *n*	67	22	26	19	24
Age at examination, median (range)	13 (5–17)	13 (5–17)	13.5 (5–17)	12 (7–17)	9.5 (2–17)
Female, *n* (%)	49 (73)	17 (77)	21 (81)	11 (58)	9 (38)
JIA subtype					
Oligoarticular, *n* (%)	24 (36)	5 (23)	10 (38)	9 (47)	–
Polyarticular, *n* (%)	31 (46)	12 (55)	13 (50)	6 (32)	–
Systemic Onset, *n* (%)	0	0	0	0	–
Psoriatic, *n* (%)	5 (8)	3 (14)	0	2 (11)	–
Enthesitis–related, *n* (%)	7 (10)	2 (8)	3 (12)	2 (10)	–
Age at diagnosis, median (range)	9 (1–15)	6.5 (15–1)	8 (2–14)	5 (3–13)	–
TMJ pain within 2 weeks of MRI, *n* (%)	22 (33)	9 (41)	12 (46)	1 (5)	–
Decreased mouth opening, <40 mm, *n* (%)	24 (36)	7 (32)	8 (31)	3 (16)	–
Crepitation, *n* (%)	10 (15)	4 (18)	5 (19)	1 (5)	–
Uveitis, *n* (%)	11 (16)	2 (9)	6 (23)	3 (16)	–
Osteochondral changes, *n* (%)	51 (76)	20 (91)	22 (85)	9 (47)	–

### Imaging

Coronal and axial MRI images of the TMJ were retrieved from our institutional PACS for each JIA patient and non-rheumatologic control. In patients with multiple MRI examinations, images from the first MRI examination within the study period were included.

JIA patients were imaged on a 3.0 Tesla scanner (Achieva, Phillips Medical Systems, Bothell, WA) with a 32-channel head coil in closed mouth (neutral) position. The following sequences were used in the rheumatologic group in this study: pre-contrast T1-weighted coronal fast spin-echo (slice thickness of 2.0 mm; gap of 2.2 mm; repetition time (TR) of 600 ms; echo time (TE) of 21 ms; bandwidth of 291; number of excitations (NEX) range of 2–4; scan time range of 3–4 min) and contrast enhanced coronal fat-saturated T1-weighted fast spin echo (slice thickness of 2.0 mm; slice gap of 2.2 mm; TR of 600 ms; TE of 20 ms; bandwidth of 292; NEX range of 2–4; scan time range of 3–4 min). The majority (>80 %) of the post contrast coronal images were acquired within 5 min after intravenous (IV) administration of gadolinium-based contrast (Gadovist, Schering Pharma, Berlin, Germany) at 0.1 ml/kg of body weight.

MR imaging of the non-rheumatologic group consisted of T1-weighted coronal fast spin-echo and post contrast coronal fat-saturated T1-weighted fast spin echo images acquired under a 1.5 T (Philips Achieva, Bothell, WA; Siemens Advanto AG, Germany; GE Twin Speed Excite, Milwaukee, WI) or 3.0 T scanner (Philips Achieva, Bothell, WA). MRI examinations of 10/24 (42 %) patients were performed under 1.5 T and 14/24 (58 %) patients were performed under 3.0 T. The MRI parameters for this group included slice thickness of 2–5 mm; slice gap of 2.5–3.5 mm; TR of 488–677 ms; TE of 9–11 ms; bandwidth of 151–346, NEX of 2–3 and scan time range of 3–4 min. The same dose and type of contrast were used for the non-rheumatologic and for the JIA patients (intravenous Gadovist at 0.1 ml/kg).

### Qualitative interpretation of MRI examinations of JIA patients

MRI examinations of the JIA group were reviewed independently and then by consensus by two experienced pediatric radiologists (J.S. and A.S.D) with over 5 and 10 years of experience in pediatric musculoskeletal imaging, respectively. The MRI examinations of TMJs in our cohort of JIA patients were categorized into one of the following categories: (1) mild active synovitis; (2) moderate/severe active synovitis; (3) no active synovitis (rheumatologic control). The subdivision of the active synovitis group into mild, and moderate/severe synovial enhancement was based on the previously published criteria by Muller et al. [11]. According to these criteria, increased joint enhancement was graded as mild when the signal of the synovial membrane on fat-saturated T1-weighted post contrast images was hyperintense to muscle and as moderate/severe when the synovial membrane was isointense to vasculature [11]. In this study, we defined mild synovitis as requiring mild synovial enhancement with or without synovial hypertrophy and moderate/severe synovitis as requiring at least moderate synovial enhancement with or without synovial hypertrophy. For a joint to be considered “without active synovitis” it would have to present with absence of both synovial enhancement and thickening. TMJs of JIA patients were also evaluated for osteochondral changes, which was defined as the presence of one or more of the following: condylar flattening, subchondral cysts or articular surface erosions. Concerning condylar enhancement, each TMJ was categorized into the same three categories based on assessment of the degree of condylar enhancement by the same pediatric radiologists.

### Quantitative interpretation

**Fig. 1:**
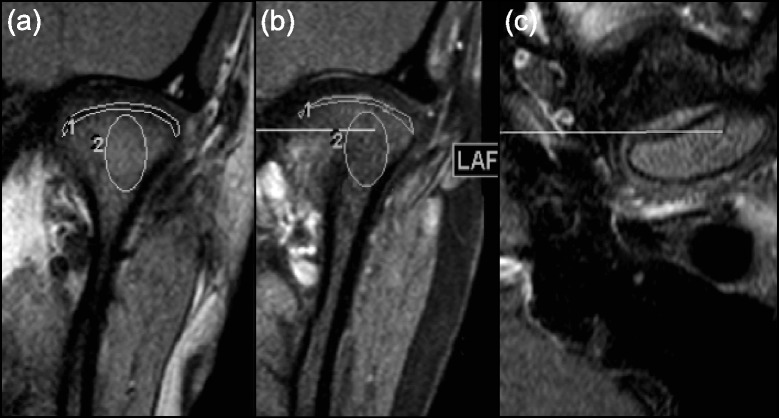
Regions of interest used to measure the synovial (1) and condylar (2) enhancement. Pre- (**a**) and post- (**b**) contrast coronal T1 weighted (W) images with the region of interest and the corresponding post contrast T1W axial image (**c**) showing that the two coronal images represent similar regions in the sagittal plane

For each TMJ, in all JIA and non-rheumatologic categories, coronal fat saturated T1-weighted post-contrast static MR images were compared to the corresponding pre-contrast coronal images (Fig. 1). To ensure that comparisons were made between similar anatomic regions, coronal pre and post contrast images were cross-referenced to the corresponding axial images using PACS software tools. This allowed us to determine the location of each coronal slice in the sagittal plane. Pre- and post-contrast coronal images were then evaluated on at least two representative slices that provided the best visualization of the synovial tissue. Using PACS software tools, a region of interest (ROI) was drawn to encompass the synovial tissue according to pre-established anatomic landmarks. The signal intensity (SI) was automatically determined from the ROI by the software. Background noise was measured using three 50 × 50 mm ROIs placed in the region of the image outside of any anatomic structures. Signal to noise ratios (SNRs) were calculated by dividing the mean signal intensity of the synovial tissue by the standard deviation of the background noise as previously described elsewhere [17, 18]. The measurements that were used to assess synovial and condylar enhancement were: enhancement ratio (ER) = (SNRpost- SNRpre)/SNRpre and relative SI change = (SIpost – SIpre)/SIpre [18, 19]. Mean SI values calculated from the two representative slices were used to determine the ER and relative SI change for each TMJ. To evaluate condylar enhancement, a circular ROI was drawn within the condyle on pre- and post-contrast coronal images (Fig. 1). The slice with the highest SI was used to calculate the ER and relative SI change to assess condylar enhancement.

SI measurements for synovial and condylar enhancement were performed by two operators (G.M. and A.A.) and were repeated by one of them (G.M.). Both individuals were blinded to whether the patient had an underlying JIA diagnosis as well as to the subjective JIA categorization for each TMJ.

### Statistical analysis

Analysis of variance (ANOVA) was used to assess the differences in means in the calculated ER and relative SI change between the four categories: non-rheumatologic control, rheumatologic control, mild active synovitis, and moderate/severe active synovitis. Student *t*-test with Dunnett adjustment for multiple comparisons was then used to determine whether there was a difference between the rheumatologic control and the remaining three groups as well as between the non-rheumatologic control (performed under 1.5 T and 3.0 T) and the JIA subgroups. Intra- and inter-observer agreement was assessed using intra-class correlation coefficients (ICCs). Statistical analysis was performed using the SAS software (SAS 9.4, SAS Institute, Cary, NC, USA). Two-tailed *P* values of < 0.05 were considered statistically significant. The Spearman’s correlation coefficient was used to determine if there is an association between the degree of synovial and condylar enhancement as well as between synovial enhancement and thickening.

## Results

### Patient characteristics

**Fig. 2:**
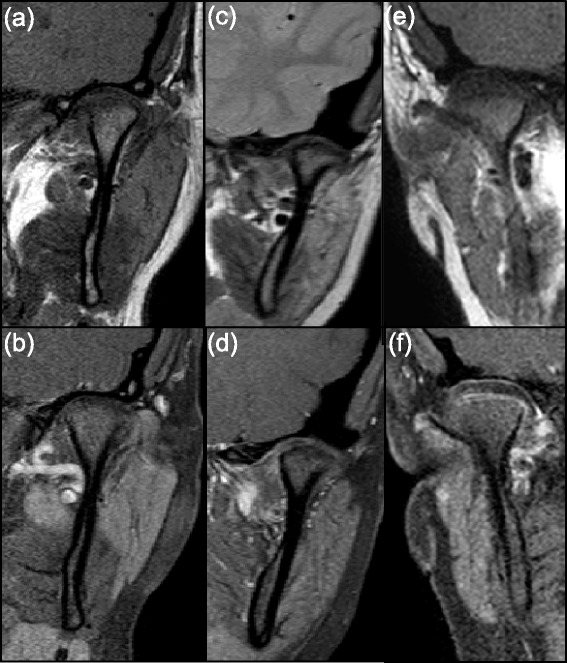
Representative images of various degrees of synovitis and condylar enhancement on qualitative assessment. Coronal T1 weighted pre- (top) and post contrast (bottom) images of TMJs with: no evidence of synovitis or condylar enhancement (**a, b**), mild synovitis without condylar enhancement (**c, d**) and moderate/severe synovitis with mild condylar enhancement (**e, f**)

**Fig. 3:**
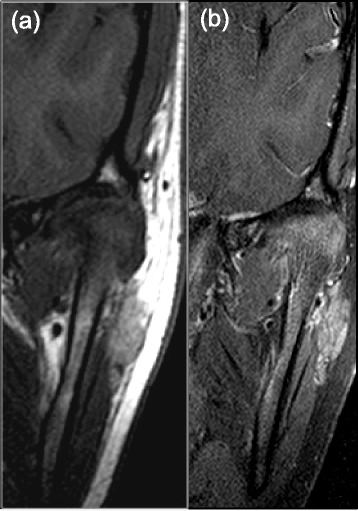
Pre- (**a**) and post- (**b**) contrast coronal T1 images of moderate/severe condylar and synovial enhancement

A total of 24 children or 48 TMJs, whose MR images completely depicted the bilateral TMJs in the coronal plane on pre-contrast T1 and post gadolinium T1 fat saturated sequences were included in the study as our non-rheumatologic control. 20/48 (42 %) TMJs were performed under 1.5 T with the remaining performed under 3.0 T. The JIA group consisted of 67 patients or 134 TMJs, which were further separated into three subgroups based on the degree of active synovitis on subjective assessment of each TMJ: mild, moderate/severe and no active synovitis (rheumatologic control). Representative images of the three subgroups are shown in Figs. 2 and 3. These TMJs were re-categorized into the same three subgroups based on visual assessment of the degree of condylar enhancement (Figs. 2 and 3). The baseline characteristics of JIA and non-JIA patients included in the study are summarized in Table 2.

In our study, 32/48 (67 %) patients with MRI findings of active arthritis (either condylar enhancement or synovitis) and 5/19 (21 %) patients with no evidence of active TMJ involvement on MRI were found to have clinical symptoms that consisted of one of the following: pain within 2 weeks of the MRI, new onset of decreased mouth opening, or crepitation. These symptoms were previously found to be suggestive of active TMJ involvement on clinical examination [6, 11, 20]. Out of the 48 patients with active TMJ arthritis, 16 (33 %) patients had active involvement of other joints, such as the knee or sacroiliac joints, within 3 months of the MRI. On the other hand, only 2/19 (11 %) patients without evidence of active TMJ involvement on MRI had recent involvement of additional joints. 37/48 (77 %) patients with active TMJ arthritis in comparison with 8/19 (42 %) patients without active TMJ arthritis had one of the following therapies within 3 months of the MRI: disease-modifying anti-rheumatic drug (DMARD), non-steroidal anti-inflammatory drug (NSAID), or TMJ steroid injection. 26/48 (54 %) patients with active TMJ arthritis was on a DMARD at the time of the MRI, while only 9 patients had a TMJ steroid injection within 3 months of the MRI. In those without active TMJ involvement, 3/19 (16 %) patients were on a DMARD and only 2 had a recent TMJ injection. However, clinical data on the presence of additional joint involvement and current therapy was unclear or unavailable in 12 (6 of which had active TMJ involvement) and 7 patients (3 of which had active TMJ arthritis), respectively. Our findings suggest an association between active TMJ arthritis and higher incidences of other joint involvement and current DMARD therapy.

### Synovial enhancement

MRI evidence of TMJ synovitis was present in 74/134 (55 %) TMJs of JIA patients on subjective assessment: 40/74 (54 %) was categorized as having mild disease, while 34/74 (46 %) was found to have moderate/severe synovitis. Our rheumatologic control consisted of the remaining 60/134 (45 %) TMJs.

TMJs of JIA patients without active disease on MRI (rheumatologic controls) were found to have a mean ER and relative SI change of 0.48 and 0.43, respectively. The non-rheumatologic control group with a mean ER of 0.09 and relative SI change of 0.66 significantly differed in terms of ER from our rheumatologic controls (*P = 0.04)*, but not in terms of relative SI change (*P = 1.00*).

**Fig. 4:**
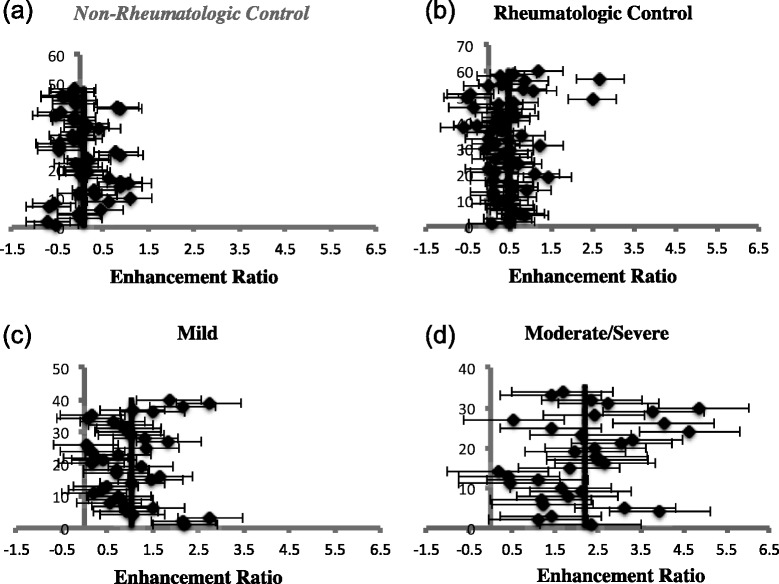
Graphic representation comparing synovial enhancement ratios of mild JIA, moderate/severe JIA, rheumatologic and non-rheumatologic controls. The enhancement ratios of the two control groups are shown in (**a**) and (**b**). The 1.5 T and 3.0 T non-rheumatologic control cases have been grouped together in this figure. TMJs with mild or moderate/severe synovitis are shown in (**c**) and (**d**), respectively. It is interesting to note that there is more variability in the range of enhancement ratios for the moderate/severe JIA group as compared to the other groups

TMJs with mild synovitis had a mean ER of 1.05, which significantly differed from both the rheumatologic and non-rheumatologic control groups with *P*-values of *<0.001* and *<0.0001*, respectively. Relative SI change for MRI examinations of TMJs with mild synovitis however, did not statistically differ from that of the two control groups, with *P = 1.00* for the non-rheumatologic control and *P = 0.10* for the rheumatologic control. These findings are summarize in Table 3 and suggest that ER, which is calculated from SNR, may allow detection of small signal intensity differences that may not be detected with relative SI change. Graphic representation comparing the synovial ER of our four groups is shown in Fig. 4.

**Table 3:** Summary of enhancement ratios and relative signal intensity changes for synovial enhancement

Enhancement ratio	Mean	95 % confidence interval	*P value (RC)*	*P value (NRC)*	*P value (NRC 1.5 T)*	*P value (NRC 3.0 T)*
Non-rheumatologic control (48)	0.090	−0.0478 – 0.227	*0.04*	-	–	–
Rheumatologic control (60)	0.480	0.335 – 0.626	–	*0.04*	*0.47*	*0.03*
Mild (40)	1.045	0.824 – 1.266	*0.001*	*<0.0001*	*0.001*	*<0.0001*
Moderate/severe (34)	2.188	1.786 – 2.577	*<0.0001*	*<0.0001*	*<0.0001*	*<0.0001*
Relative signal intensity change	Mean	95 % confidence interval	*P value (RC)*	*P value (NRC)*	*P value (NRC 1.5 T)*	*P value (NRC 3.0 T)*
Non–rheumatologic control (48)	0.660	0.401 – 0.919	*1.00*	–	–	–
Rheumatologic control (60)	0.433	0.229 – 0.638	–	*1.00*	*1.00*	*0.18*
Mild (40)	0.919	0.640 – 1.197	*0.10*	*1.00*	*0.20*	*1.00*
Moderate/severe (34)	1.953	1.487 – 2.420	*<0.0001*	*<0.0001*	*<0.0001*	*<0.0001*

The *P* values represent comparisons between TMJs with mild or moderate/severe synovial enhancement (based on qualitative assessment) and the results from the rheumatologic (RC) and non-rheumatologic control groups (NRC). The non-rheumatologic control group was further separated into MRI examinations that were performed under 1.5 T and 3.0 T. Further comparison is made between the two subgroups and the TMJs of JIA patients. The number in () represents the number of TMJs in each group

As our non-rheumatologic control consisted of MRI examinations performed under 1.5 T [20/48 TMJs (42 %)] and 3.0 T [28/48 TMJs (58 %)], subgroup analysis was performed to determine whether there is a difference between the examinations performed at the two magnetic field strengths. The examinations performed at 1.5 T did not significantly differ from those performed at 3.0 T with regards to ER and relative SI change (*P > 0.05)*. Separate comparisons between the non-rheumatologic controls performed at each of the two magnetic field strengths (1.5 T and 3.0 T) with TMJs demonstrating mild synovitis again showed significant difference in terms of ER (*P < 0.001*) but not in terms of relative SI change (*P > 0.20*) (Table 3).

**Fig. 5:**
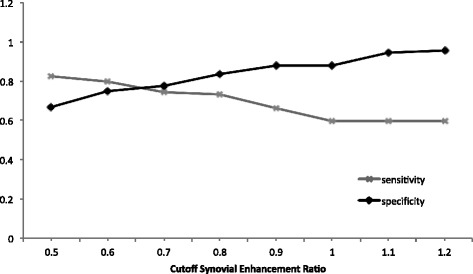
Various cutoff synovial enhancement ratios and their sensitivities and specificities

As ER appeared to be a better predictor of active disease in comparison with relative SI change, the mean synovial ER of 1.0 for TMJs with mild synovitis was tested as the cutoff value for distinction between TMJs with at least mild synovitis on MRI from those without. This yielded a sensitivity of 0.59 and a specificity of 0.88, when used on the ERs from our four groups. Additional cutoff values that were evaluated along with their sensitivities and specificities are summarized in Fig. 5.

The inter-operator and intra-operator reliabilities for our quantitative analysis of synovial enhancement were excellent for both ER (ICC = 0.93 and 0.97, respectively) and relative SI change (ICC = 0.98 and 0.98, respectively).

### Condylar enhancement

On consensus subjective assessment, 68 out of 134 (51 %) TMJs were found to have at least mild condylar enhancement (53 [40 %] mild and 15 [11 %] moderate/severe). The rheumatologic control group consisted of the remaining 66/134 (49 %) TMJs that did not demonstrate any bone marrow enhancement on subjective assessment. The mean ER and relative SI change of the rheumatologic control group were 0.112 and 0.008, respectively. Although the two control groups did not significantly differ in terms of ER (*P = 1.00*), they did differed in terms of relative SI change with *P < 0.0001* (Table 4). We suspect that this may be related to slight differences in technique between the two controls, as relative SI change does not account for differences in background noise related to differences in technique; whereas, background noise is included in the calculation of ER.

**Table 4:** Summary of enhancement ratios and relative signal intensity changes for condylar enhancement

Enhancement ratio	Mean	95 % confidence interval	*P value (RC)*	*P value (NRC)*	*P value (NRC 1.5 T)*	*P value (NRC 3.0 T)*
Non-rheumatologic control (48)	0.138	−0.063 – 0.339	*1.00*	-	–	–
Rheumatologic control (66)	0.112	0.011 – 0.213	–	*1.00*	*0.06*	*0.16*
Mild (53)	0.592	0.380 – 0.804	*0.0008*	*0.005*	*0.96*	*<0.0001*
Moderate/severe (15)	1.284	0.827 – 1.740	*<0.0001*	*<0.0001*	*0.007*	*<0.0001*
Relative signal intensity change	Mean	95 % confidence interval	*P value (RC)*	*P value (NRC)*	*P value (NRC 1.5 T)*	*P value (NRC 3.0 T)*
Non–rheumatologic control (48)	0.588	0.391 – 0.785	*<0.0001*	–	–	–
Rheumatologic control (66)	0.008	−0.097 – 0.113	–	*<0.0001*	*0.01*	*<0.0001*
Mild (53)	0.470	0.267 – 0.673	*0.001*	*1.00*	*1.00*	*0.26*
Moderate/severe (15)	0.951	0.542 – 1.360	*<0.0001*	*0.35*	*0.07*	*0.51*

The *P* values represent comparisons between TMJs with mild or moderate/severe condylar enhancement (based on qualitative assessment) and the results from the rheumatologic (RC) and non-rheumatologic control groups (NRC). The non-rheumatologic control group was further separated into MRI examinations that were performed under 1.5 T and 3.0 T. Further comparison is made between the two subgroups and the TMJs of JIA patients. The number in () represents the number of TMJs in each group

**Fig. 6:**
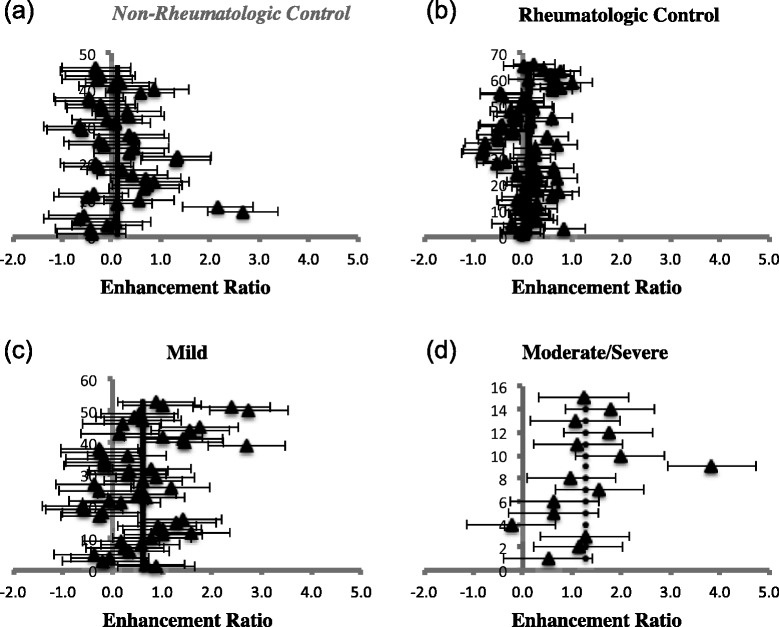
Graphic representation comparing condylar enhancement ratios of the same four groups as Fig. 4. The enhancement ratios of the two control groups are shown in (**a**) and (**b**). TMJs with mild or moderate/severe condylar enhancement are shown in (**c**) and (**d**), respectively. Excluding the outlier for the moderate/severe JIA group concerning condylar enhancement, there is less variability in the range of condylar enhancement ratios for the moderate/severe JIA group compared to synovial enhancement ratios for the same JIA group (Fig. 4)

TMJ MRI examinations with mild condylar enhancement had a mean ER of 0.592, which differed significantly from both control groups (*P < 0.005*). In terms of relative SI change, TMJs with mild condylar enhancement significantly differed from that of the rheumatologic controls (*P = 0.001*) but were found to have a mean that was less than that of the non-rheumatologic control (0.470 vs 0.588). However, this was calculated to be not statistically significant (*P = 1.00*). Similar observations were seen for relative SI change of TMJs with moderate/severe condylar enhancement, which significantly differed from only the rheumatologic control (*P < 0.0001*), but not the non-rheumatologic control (*P = 0.35*) (Table 4). These findings further suggest that ER may be a better quantitative measure of enhancement in comparison with relative SI change. Graphic representation comparing the condylar ER of our four groups is shown in Fig. 6.

Subgroup analysis comparing the ER of non-rheumatologic control examinations performed at 1.5 T and 3.0 T with TMJs demonstrating mild condylar enhancement showed that only examinations performed at 3.0 T were significantly different (*P < 0.0001)*. In terms of relative SI change, non-rheumatologic control examinations performed at each of the two magnetic field strengths again did not significantly differ from those with mild and moderate condylar enhancement (*P > 0.07*) (Table 4).

**Fig. 7:**
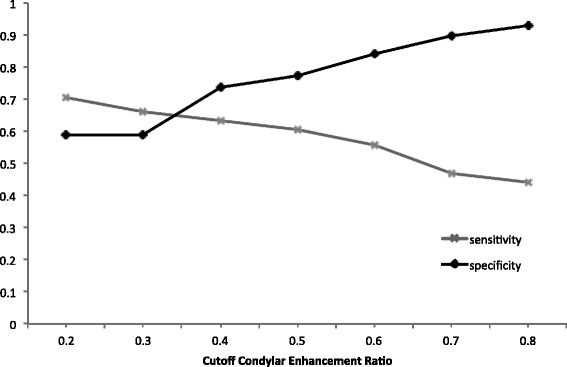
Various cutoff condylar enhancement ratios and their sensitivities and specificities

Similar to the analysis for synovial enhancement, the mean condylar ER of 0.6 for TMJs with mild condylar enhancement was tested as the cutoff value for distinction between TMJs with at least mild condylar enhancement on MRI from those without, yielding a sensitivity of 0.55 and a specificity of 0.84. Additional cutoff values that were evaluated along with their sensitivities and specificities are summarized in Fig. 7.

The inter-operator and intra-operator reliabilities for our quantitative analysis of condylar enhancement were excellent for both ER (ICC = 0.93 and 0.98, respectively) and relative SI change (ICC = 0.96 and 0.94, respectively).

### Association between morphologic changes, synovial and condylar enhancement

Osteochondral changes were present in the majority (>80 %) of TMJs with active synovitis on qualitative assessment compared with our rheumatologic control of 45 % as summarized in Table 5. The mean synovial thickness was found to progressively increase with the severity of synovial enhancement. The mean synovial thickness of TMJs with mild and moderate/severe synovial enhancement differed significantly from those of the rheumatologic control (*P < 0.0001*). The Spearman’s correlation coefficient demonstrated negative association between the synovial thickness and ER for synovial enhancement in the rheumatologic control but mild to moderate positive association in those with active synovitis. However, 8/40 (20 %) with mild synovitis and 2/34 (6 %) with moderate/severe synovitis did not demonstrate any synovial hypertrophy on MRI.

**Table 5:** Association between degree of synovitis and presence of osteochondral changes, synovial thickening, and enhancement ratios

	Osteochondral changes, N (%)	Synovial thickening, mean, mm	ER for synovial enhancement, mean	Correlation between synovial ER and thickening	ER for condylar enhancement, mean	Correlation between synovial and condylar ER
Rheumatologic control *N* = 60	27 (45)	1.1	0.480	−0.220	0.148	0.329
Mild *N* = 40	32 (80)	1.9	1.045	0.335	0.396	0.443
Moderate/Severe *N* = 34	31 (91)	3.2	2.188	0.400	0.983	0.379

Osteochondral changes are defined as the presence of at least one of the following: condylar flattening, subchondral cysts or articular surface erosions. The correlation between synovial enhancement ratios and synovial thickening as well as condylar enhancement ratios were calculated using the Spearman’s correlation coefficient for each subjective category of synovitis. N represents the number of TMJs

Similarly, progressively higher condylar enhancement (in terms of ER) was seen in TMJs with mild and moderate/severe synovial enhancement compared with those without synovial enhancement on MRI. The Spearman’s correlation coefficient showed mild to moderate positive association between the ER of synovial and condylar enhancement. 16/40 (40 %) TMJs with mild synovitis and 11/34 (32 %) with moderate/severe synovitis did not demonstrate any condylar enhancement on the qualitative assessment (Table 5).

## Discussion

Previous studies have shown the value of contrast-enhanced MRI in the assessment of the synovium and mandibular condyle, allowing early depiction of TMJ changes such as synovial and condylar enhancement before significant osseous destruction and symptoms occur [15, 21, 22]. However, accurate distinction between JIA patients with mild early arthritis from those without active disease can be challenging. Our results demonstrated that the quantitative SNR measure, ER, varied proportionately with the degree of synovitis and condylar enhancement seen on contrast enhanced MRI, suggesting that specific ER values may be useful as cutoffs to aid in distinguishing TMJs with mild involvement (in terms of synovial and condylar enhancement) from those without evidence of active disease on MRI. We propose that the synovial ER threshold of 0.6 with a sensitivity of 80 % and specificity of 75 % may be a reliable predictor of the presence of active synovitis. This method of quantitative TMJ assessment had excellent inter- and intra-reader reproducibility.

There is currently a controversy regarding whether any synovial or condylar enhancement is pathologic or physiologic in children. Although previous studies have regarded any enhancement to be pathological in children and adults [2, 3, 21, 23], in our study TMJs without active arthritis were found to have some degree of synovial enhancement on signal to noise and signal intensity measurements. Our criteria for the definition of TMJs that do not present with active synovitis was based on the previously published criteria by Muller et al. [11], where the synovium must be hypointense to muscle on fat saturated T1 post contrast images and without evidence of synovial hypertrophy [11]. Similarly, condyles that did not have any evidence of enhancement on qualitative analysis were also found to have some degree of enhancement on the quantitative analysis. These findings are similar to the results from the recent study by Von Kalle et al. [12], where quantitative analysis of TMJs in children who had MRI performed for reasons other than TMJ disease were found to have both synovial and condylar enhancement.

Although previous literature [14] has evaluated active TMJ involvement based on the presence of synovial thickening and osteochondral changes (condylar flattening, articular surface erosions or subchondral cysts), assessment of the chronicity of these findings is challenging as both may occur in the acute and chronic stages of TMJ disease in JIA. Whereas osteochondral changes may be related to previous episodes of TMJ inflammation, synovial thickening may be secondary to chronic pannus formation. In our population, even though osteochondral changes were more prevalent in TMJs with synovitis, many TMJs that did not have active TMJ involvement on qualitative assessment were found to have osteochondral changes. In addition, despite our results demonstrating a positive correlation between the degree of synovial thickness and synovial enhancement measured both qualitatively and in terms of ER, in our study 20 % of TMJs with mild synovitis on visual assessment (based on the previously published criteria by Muller et al. [11]) lacked synovial hypertrophy. Therefore, osteochondral changes and synovial thickening may not be reliable indicators of active disease and should be differentiated from synovial enhancement when evaluating for active TMJ involvement in JIA patients to determine the need for treatment. This distinction between synovial thickening and enhancement aligns with previous literature by Vaid et al., and reiterates the importance of synovial enhancement as the most common early finding of TMJ involvement in children with JIA [3, 4, 11, 15, 23, 24].

Similarly, TMJs with a greater degree of synovial enhancement were associated with higher mean condylar enhancement ratios. However, a large proportion of our population (36 %) with synovial enhancement did not demonstrate any condylar enhancement on qualitative assessment, suggesting that the absence of condylar enhancement does not necessarily exclude the presence of active TMJ inflammation.

In agreement with prior studies, clinical symptoms (new onset of TMJ pain, decreased mouth opening or crepitation) were found to be poor predictors of the presence or absence of TMJ involvement demonstrated on the subsequent MRI examination in our cohort of JIA patients [4, 6, 10, 11, 20]. A prior study by Weiss et al. demonstrated that TMJ involvement was highly prevalent at the time of JIA diagnosis despite lack of clinical symptoms [4]. Our observations that active TMJ involvement on MRI were associated with higher incidences of recent involvement of other joints, suggest that further evaluation on the correlation between active TMJ and other joint arthritis may be helpful in directing clinical management. These findings further re-emphasize the importance of diagnosing TMJ involvement with imaging in children with JIA.

The chief limitation in our study is that pre-contrast coronal T1 images were obtained without fat saturation, whereas post-contrast images were obtained with fat saturation, as this is part of our routine protocol. In the presence of fat suppression, there is loss of the intrinsic T1 high signal intensity from the fatty components within the bone marrow, resulting in a lower SI in the post contrast images compared to similar non-fat-saturated images [25]. As a result, we anticipate that this difference between the pre- and post-contrast images may have underestimated the ER and relative SI change for condylar enhancement.

Due to the retrospective nature of this study, MRI examinations of the non-rheumatologic group had slight differences in technique, specifically the TR, TE and magnetic field strengths (both 1.5 T and 3.0 T) used during the scan, compared with that of the JIA and rheumatologic control groups (3.0 T only). Therefore, a quantitative measure that uses SNR, such as ER, would be expected to be a better measure for comparing our JIA groups with MRI images of the non-rheumatologic control (in comparison with measures that uses only the SI such as relative SI change) [26, 27]. Since relative SI change does not take into account the random background variations in signal (noise) overlying anatomic structures, it does not adjust for differences in noise due to the presence or absence of fat saturation in the post and pre-contrast images or slight differences in technique between the JIA patients and the non-rheumatologic control. Consequently, relative SI change may not be able to detect small differences in SI as was demonstrated by our results comparing those with mild disease to the two control groups [26, 27]. Such findings on static contrast-enhanced MR images align with those of Von Kalle et al. [14], which were obtained on dynamic contrast enhanced MRI in the axial plane at 1.5 T (in contrast to our study, where the images were obtained in the coronal plane at 3.0 T). The results of both studies demonstrate that the degree of contrast enhancement calculated using relative SI change, (SIpost – SIpre)/SIpre, regardless of being static or dynamic, may not allow for accurate differentiation between TMJs with and without synovitis. On the other hand, our results demonstrated that a quantitative measure that takes into account the background noise (specifically ER) may be a good measure for assessing the degree of enhancement in TMJs, particularly when comparing different sequences (as is often the case when comparing pre- and post contrast images from MRI examinations of the TMJ in patients with JIA) [27].

An additional limitation related to the retrospective nature of the study was the fact that because the MRI examinations of the study were selected through an imaging database rather than consecutively through a systematic monitoring system, selection bias should be considered at some extent.

Furthermore, although reviewers were blinded to the subjective groupings of the JIA MRIs and to whether or not patients had an underlying diagnosis of JIA, differences in technique in the non-rheumatologic control could have introduced an undesirable “unblinding” component to the review of examinations based on the appearance of the images. However, all quantitative measurements were made according to pre-established anatomic landmarks and a second control group using the unaffected TMJs of JIA patients was also used for comparison.

Finally, comparisons between the ER of our non-rheumatologic and rheumatologic controls showed that ER differed significantly in terms of synovial enhancement but not for condylar enhancement. The mean synovial ER for the non-rheumatologic control was significantly lower than that of the rheumatologic control. Nevertheless, the contrast enhanced coronal sequences of the majority (>80 %) of our JIA patients were acquired within 5 min after gadolinium administration, while in the non-rheumatologic controls, the duration of delay for coronal images was longer, with the images completed at >8 min after contrast injection. Therefore, concerning comparative assessment where no major measurement bias is expected between the rheumatologic and non-rheumatologic control TMJs, such a bias is expected between the two groups in our study due to the differences in timing of the post-contrast coronal images. To our knowledge, there is limited published literature that evaluates variation of contrast enhancement with time, particularly at greater than 6 min after contrast injection. A prior study by Yamato et al. demonstrated that synovial enhancement in the knee was a time dependent phenomenon with an optimal imaging time of less than 5mins following contrast administration [28]. It is likely that some diffusion of contrast material into the adjacent joint fluid had already occurred at the time coronal images were acquired in the non-rheumatologic controls, leading to the discrepancy in synovial ERs between the two control groups [26, 28]. Condylar enhancement may be less sensitive to the timing of image acquisition or retain contrast material for a longer duration; however, optimal imaging time for condylar enhancement has rarely been published.

Given these limitations, the study design incorporated two control groups: TMJs without active involvement in JIA patients and TMJs of patients without any rheumatologic disease who had MRI of the brain for other reasons. Comparison of findings between the TMJs with active involvement and those of the rheumatologic control group was made using data acquired with the same MRI protocol and field strength (3.0 T). Although not all non-rheumatologic control cases were obtained at 3.0 T, a direct comparison of findings between the JIA and non-rheumatologic control groups at the same MRI field strength was possible in 58 % of the non-rheumatologic control cases. Nevertheless, the ER and relative SI change of examinations performed at 1.5 T and 3.0 T were not significantly different. The results from comparisons made with the non-rheumatologic control cases performed under 1.5 T were similar to those of the 3.0 T, with the exception of subgroup analysis comparing ER of mild condylar enhancement with the non-rheumatologic cases performed at 1.5 T. The ability to detect a difference between TMJs with mild condylar enhancement and the non-rheumatologic cases at 3.0 T but not at 1.5 T is likely related to inadequate statistical power due to the smaller sample size that resulted when the non-rheumatologic control cases were separated into the two subgroups.

## Conclusion

Quantitative signal to noise ratios of the degree of temporomandibular joint synovial and condylar marrow enhancement using static MRI in the coronal plane generate thresholds and tendencies and offer an additional tool in conjunction with subjective visual assessment to aid in the diagnosis of mild active temporomandibular joint involvement. Such quantitative measures may also allow us to more accurately compare imaging findings to assess responses to treatments and help us to direct further interventions, as needed, for children and adolescents with juvenile idiopathic arthritis. In addition, osteochondral changes and synovial thickening may not be reliable indicators of active disease and should be differentiated from synovial enhancement, as discussed in previous literature [15], when evaluating for active TMJ involvement in JIA patients to determine the need for treatment. This re-iterates the importance of accurately distinguishing TMJs with mild synovial enhancement from those without.
